# Homozygous C1qA Deficiency Presenting as Early‐Onset Systemic Lupus Erythematosus: A Case Report With a Literature Review

**DOI:** 10.1155/crii/4667037

**Published:** 2026-05-14

**Authors:** Hadi Mottaghipisheh, Andisheh Mosaffa Jahromi, Fatemeh Mirzaei, Seppo Meri, Kurosh Kalantar

**Affiliations:** ^1^ Hematology Research Center, Shiraz University of Medical Sciences, Shiraz, Iran, sums.ac.ir; ^2^ Department of Immunology, School of Medicine, Shiraz University of Medical Sciences, Shiraz, Iran, sums.ac.ir; ^3^ Department of Bacteriology and Immunology, Translational Immunology Research Program (TRIMM), University of Helsinki and HUSLAB, Helsinki University Hospital, Helsinki, Finland, hus.fi; ^4^ Autoimmune Diseases Research Center, Shiraz University of Medical Sciences, Shiraz, Iran, sums.ac.ir

**Keywords:** autoimmunity, C1qA, complement, systemic lupus erythematosus (SLE)

## Abstract

**Background:**

Childhood‐onset systemic lupus erythematosus (cSLE) can be particularly severe when associated with inborn errors of immunity. Hereditary C1q deficiency is an exceptionally rare complement disorder that confers a very high risk of early, aggressive lupus. Reporting this case highlights the need to consider complement deficiencies in young children presenting with lupus‐like features and recurrent infections and illustrates the diagnostic value of integrating immunologic and genetic testing.

**Case Presentation:**

A 3‐year‐old girl presented with recurrent infections, a photosensitive rash, and persistent leukopenia, raising clinical suspicion for an underlying complement deficiency in the context of cSLE. Laboratory evaluation showed strongly positive antinuclear antibody (ANA) and anti‐SSA antibodies, with normal C3 and C4 levels but markedly reduced CH50. Lymphocyte subset analysis by flow cytometry was unremarkable. Western blot analysis and whole‐exome sequencing identified a homozygous nonsense variant in C1qA (c.622C > *T*; p.Gln208 ^∗^), confirming complete hereditary C1q deficiency. Despite treatment with corticosteroids and hydroxychloroquine, the patient’s disease activity remained suboptimal, leading to consideration of hematopoietic stem cell transplantation as a definitive therapeutic option.

**Conclusions:**

This case emphasizes the importance of early recognition and systematic evaluation of complement deficiencies in pediatric patients with lupus manifestations and recurrent infections. Identifying hereditary C1q deficiency has crucial implications for diagnosis, prognosis, and timely selection of advanced therapies, including hematopoietic stem cell transplantation, in severe forms of cSLE.

## 1. Introduction

Childhood‐onset systemic lupus erythematosus (cSLE) is a multisystem autoimmune disorder whose severity correlates with the extent of organ involvement [1]. Earlier disease manifestation is associated with more aggressive phenotypes, including increased rates of proteinuria, malar rash, arthritis, anemia, and leukopenia. Emerging evidence links cSLE to underlying inborn errors of immunity, highlighting potential genetic predispositions in disease pathogenesis [2]. In particular, defects in the classical complement pathway have long been linked to the pathogenesis of SLE [3]. C1q is best known as the recognition molecule that initiates the classical complement pathway. It is a serum protein composed of six heterotrimeric subunits. Each subunit has three polypeptide chains (C1qA, C1qB, and C1qC), whose C‐termini constitute globular domains that act as the recognition units of the C1q complex. It is unique among complement proteins in being primarily produced by monocyte‐lineage cells rather than by the liver. Additionally, C1q can modulate immune cell function, promoting the noninflammatory uptake of apoptotic debris by macrophages. Through these actions, C1q serves as a guardian against autoimmunity [4, 5].

Hereditary C1q deficiency, caused by biallelic loss‐of‐function mutations in any of the *C1q* subunit genes, is extremely rare but carries an extraordinary predisposition to lupus. Clinical reports consistently describe a predominance of cutaneous lupus features in these patients. C1q‐deficient patients often have unusual serological profiles, high‐titer antinuclear antibodies, despite normal C3 and C4 levels. Sometimes typical anti‐double‐stranded DNA (anti‐dsDNA) antibodies are absent, reflecting the unique immunological context of lupus with absent complement activity [1, 3, 6]. Clinically, the C1q‐deficient patients can be challenging to treat. Standard immunosuppressants like corticosteroids and hydroxychloroquine often provide only partial control, as the underlying complement defect continues to fuel immune complex accumulation. In some reported cases, approaches such as regular infusions of fresh frozen plasma or even hematopoietic stem cell transplantation have been explored, highlighting the need for outside‐the‐box strategies when confronting a fixed genetic complement deficiency [7, 8]. Here, we report the clinical, immunological, and genetic findings of a 3‐year‐old girl diagnosed with C1q deficiency‐associated SLE. The diagnosis was supported by flow cytometric analysis of immune cell subsets and next‐generation sequencing (NGS), identifying a pathogenic variant in the *C1qA* gene. For further investigation of the immunological profile, the presence of C1q proteins in serum was also assessed by Western blot analysis.

## 2. Patient Information

The patient is a 3‐year‐old Iranian girl presenting with clinical features suggestive of early‐onset systemic lupus erythematosus. Before blood collection, written informed consent was obtained from the patient’s parents, who are the legal guardians. This study received approval from the Shiraz University Ethics Committee (Number 33359). A total of 5 mL of peripheral blood was collected into an EDTA tube for PBMC isolation. Additionally, 3 mL was collected into a clot‐activator tube for serum preparation and 2 mL into a citrate tube for plasma separation. Serum and plasma samples from a healthy control of the same age and sex were used as controls.

## 3. Clinical Findings

### 3.1. Hematological and Biochemical Analysis

A Complete blood count (CBC), including white blood cell (WBC), red blood cell (RBC), hemoglobin, hematocrit, and platelet counts with a full differential analysis of blood cells, was performed using a Sysmex Automated Hematology Analyzer (Sysmex Corporation, Japan) following standard hematological protocols. Liver function tests (aspartate aminotransferase [AST] and alanine aminotransferase [ALT]) and renal function tests (blood urea nitrogen [BUN] and creatinine) were conducted using a Beckman Coulter Automated Biochemistry Analyzer (Beckman Coulter, Inc., USA), adhering to standard clinical laboratory procedures (Table 1).

**Table 1:** Summary of the patient’s immunological findings and routine laboratory results compared with normal values.

The patient’s routine laboratory findings			
Category	Test	Patient value	Normal range
Blood count	WBC (×10^3^/µL)	3.81	8.0–16.0
	RBC (×10^6^/µL)	4.74	3.9–5.1
	Hemoglobin (Hb, g/dL)	12.2	11.1–14.1
	Hematocrit (HCT, %)	37.1	30.0–38.0
	Platelet count (PLT,×10^3^/µL)	241	200–550
	Neut (%)	26.6	25–50
	Lymph (%)	61.5	35–65
	Mono (%)	10.9	3–12
	Eosin (%)	0.8	0.5–5
	Basophil (%)	0.2	0.5–2
Serum chemistry	AST (U/L)	41	Up to 30
	ALT (U/L)	47	Up to 30
	BUN (mg/dL)	9.0	5–17
	Creatinine (Cr, mg/dL)	0.37	0.17–0.41

Immunological data			
Inflammatory markers	CRP (mg/L)	<2.0	Up to 6
	ESR (IU/mL)	55.2	Less than 14
Immunoglobulins	IgG (g/L)	8.20	4.53–9.16
	IgA (g/L)	0.60	0.22–1.18
	IgM (g/L)	1.35	0.19–1.50
	IgE (IU/mL)	47.2	<50
	C3 (g/L)	1.35	0.9–1.8
	C4 (g/L)	0.24	0.1–0.4
Complement data	C1q (µg/mL)	16.1	Normal: 0–16 Borderline: 16–18 Abnormal: >18
	CH50 (U/mL)	<30	50–150
Autoantibodies	ANA(Index)	3.0	Negative: <0.80
	Anti‐dsDNA (IU/mL)	2.7	Normal: <20
	Anti‐CENP Ab	3	Negative: 0–5
	Anti‐Ro (SSA)	89	Negative: 0–5 Borderline: 6–11 Positive: 11–50 Critical: >50
	Anti‐La (SSB)	3	Negative: 0–5
	Anti‐RNP/Sm	8	Negative: 0–5
	RF (IU/ml)	133	Less than 14
	ANCA‐p(U/mL)	0.3	Normal <12
	ANCA‐c(U/mL)	0.6	Normal <12
	Anti Scl70	1	Negative: 0–5
	Anti‐smith	7	Negative: 0–5
	Anti‐β2‐Glycoprotein I‐IgG (U/mL)	5.62	Negative: <12
	Anti‐β2‐Glycoprotein I‐IgM (U/mL)	0.8	Negative: <12
	Anti‐Cardiolipin Ab‐IgG(U/mL)	2.6	Negative: <12
	Anti‐Cardiolipin Ab‐IgM(U/mL)	2.11	Negative: <12
	Anti‐Jo1	0	Negative: 0–5

### 3.2. Serological Findings

Inflammatory markers, including C‐reactive protein (CRP), were quantified using biochemical assays on a Beckman Coulter Automated Biochemistry Analyzer (Beckman Coulter, Inc., USA), following standard clinical protocols. Erythrocyte sedimentation rate (ESR) was determined using the Westergren method. Serum immunoglobulins (IgG, IgA, IgM, and IgE) were measured using biochemical assays on the Beckman Coulter Automated Biochemistry Analyzer (Beckman Coulter, Inc., USA), according to established laboratory procedures. The autoantibody profile, including antinuclear antibody (ANA), anti‐dsDNA, anti‐centromere protein (anti‐CENP), anti‐Ro (SSA), anti‐La (SSB), anti‐ribonucleoprotein/Smith (anti‐RNP/Sm), rheumatoid factor (RF), anti‐neutrophil cytoplasmic antibodies (ANCA‐p and ANCA‐c), anti‐Scl‐70, anti‐Smith, anti‐β2‐glycoprotein I (IgG and IgM), anti‐cardiolipin (IgG and IgM), and anti‐Jo‐1, was assessed using enzyme‐linked immunosorbent assay (ELISA) with commercial kits from Generic Assays (Medipan GmbH, Germany), following the manufacturer’s instructions and standard serological protocols (Table 1).

### 3.3. Immunological Findings

Peripheral blood mononuclear cells (PBMCs) were separated from whole blood samples using Ficoll‐Paque density gradient centrifugation (GE Healthcare, Uppsala, Sweden). Briefly, 10 mL of peripheral blood, diluted 1:1 with phosphate‐buffered saline (PBS), was taken from the patient and added over 5 mL of Ficoll‐Paque, then centrifuged at 400 × *g* for 30 min at room temperature (RT). Following collection, the PBMC layer was washed with PBS and resuspended into RPMI‐1640 medium (Gibco, Thermo Fisher Scientific, Waltham, MA, USA) supplemented with 10% fetal bovine serum (FBS). Using a hemocytometer and the trypan blue exclusion method, cell viability and counting were carried out to ensure >95% viability before staining. In order to characterize immune cell types for immunophenotyping, PBMCs were stained using a panel of fluorochrome‐conjugated monoclonal antibodies that target surface markers. The antibodies used were from BD Biosciences, San Jose, CA, USA, and included anti‐CD3 (FITC), anti‐CD4 (PE), anti‐CD5 (APC), anti‐CD8 (PerCP), anti‐CD16 (PE‐Cy7), anti‐CD19 (APC), anti‐CD20 (FITC), anti‐CD23 (PE), anti‐CD34 (APC), anti‐CD45 (PerCP‐Cy5.5), and anti‐CD56 (PE‐Cy7). The staining solution (PBS with 2% FBS and 0.1% sodium azide) was used to resuspend about 1 × 10^6^ cells in the sample. The samples were then treated with the corresponding antibody cocktail for 30 min at 4°C in the dark. To account for nonspecific binding, isotype controls were incorporated. After incubation, cells were washed twice with staining buffer to remove unbound antibodies and resuspended in 500 µL of staining buffer for analysis. A FACSCalibur flow cytometer (BD Biosciences) was used for the flow cytometry procedure. For each tube, at least 10,000 events were collected. Single‐stained controls were used as compensation to account for fluorochrome spectrum overlap. CellQuest Pro software (BD Biosciences) was used to collect the data. FlowJo software (version 10.8, FlowJo LLC, Ashland, OR, USA) was used to analyze the flow cytometry data. After identifying lymphocyte populations using forward scatter (FSC) and side scatter (SSC), gating strategies were developed to measure the frequencies of B cells (CD19+CD20+), T‐helper cells (CD3+CD4+), cytotoxic T cells (CD3+CD8+), NK cells (CD16+CD56+), and blast cells (CD34+) using specific marker gating. Hoffman Hematology: Basic Principles and Practice, 7th Edition (2018) reference ranges were used to verify the accuracy and consistency of the results.

### 3.4. Next Generation Sequencing (NGS)

Using the QIAamp DNA Blood Mini Kit (Qiagen, Germany), genomic DNA was extracted from 5 mL of peripheral blood in accordance with the manufacturer’s instructions. A Nanodrop spectrophotometer and agarose gel electrophoresis were used to assess the quality and amount of the DNA, which had a 260/280 ratio of 1.8–2.0 and a sufficient yield for sequencing.

The Illumina NovaSeq6000 platform was used to carry out whole exome sequencing (WES). Using the Sure Select Human All Exon V7 kit (Agilent Technologies), libraries were created by enriching exonic and specific noncoding regions. The Qubit and Bioanalyzer were used to measure fragment size and library concentration, respectively. Over 95% of the target regions were highly sensitively covered by the 100 bp reads produced by paired‐end sequencing, which had an average coverage of 100x.

Raw data were processed using the Illumina DRAGEN pipeline for alignment to the human reference genome (GRCh38). Variant calling was done using GATK and ANNOVAR to identify SNVs, indels and small duplications. Variants were annotated using databases such as ClinVar, gnomAD, and 1000 Genomes. Quality control ensured reliable mapping and coverage.

### 3.5. Complement Quantification

Complement components C3 and C4 were quantified using a Prestige automated biochemistry analyzer (Tokyo Boeki Medical System, Japan) according to standard clinical laboratory protocols. Total hemolytic complement activity (CH50) was determined using a standard hemolytic assay, following established procedures. Serum levels of C1q were quantified using a commercial ELISA kit (Generic Assays, Medipan GmbH, Germany), following the manufacturer’s instructions. Additionally, the presence of the C1q protein was assessed using Western blot analysis, as detailed in the SDS‐PAGE and Immunoblotting section.

### 3.6. SDS‐PAGE and Immunoblotting

Serum and plasma from the patient and control human were collected and processed through centrifugation to eliminate cellular debris. Protein concentrations were quantified using a BCA assay to standardize the loading amounts. Equal protein quantities were applied with and without reducing agents. 50 µg amounts were resuspended in 10 μL of the loading dye solution. Equal protein quantities were applied with the reducing agent dithiothreitol (Invitrogen Blot TM U.S.A). 50 µg amounts were resuspended in 10 μL of the loading dye solution. Proteins were separated using 4%–12% Bis‐Tris gradient gels (NW04120BOX, Thermo Fisher Scientific, U.S.A). For immunoblotting, proteins were transferred to a nitrocellulose membrane (IB23001, Thermo Fisher Scientific) using iBlot 2 (Life Technologies, U.S.A). Nonspecific binding was prevented with a blocking buffer containing 5% (w/v) nonfat dry milk in PBS (137 mM NaCl, 2.7 mM KCl, 8 mM Na_2_HPO_4_, and 2 mM KH_2_PO_4_, pH 7.4) containing 0.05% Tween 20 for 1 h at RT. Polyclonal rabbit anti‐human C1q (Dako A0136) antibodies were diluted 1:10000 and deposited onto individual membrane strips in blocking buffer and incubated at 4°C for 16 h under shaking conditions. The strips were washed three times in PBS with 0.05% Tween 20 (PBS‐T) at RT, followed by a 10‐min wash at 4°C under shaking conditions. Bound IgG was detected using peroxidase‐conjugated AffiniPure goat anti‐rabbit IgG (H&L; Jackson ImmunoResearch 111‐034‐144) diluted 1:10000 in blocking buffer and incubated at RT for 1 h. After washing, the membrane was incubated in the enhanced chemiluminescence substrate containing 1 M Tris‐HCl, pH 8.5; 250 mM luminol; 90 mM coumaric acid and 30% H_2_O_2_. The resulting reactions were captured on X‐ray films (Fujifilm, Tokyo, Japan).

### 3.7. Diagnostic Assessment

The patient was a 3‐year‐old girl, who was sent to the clinic as a suspected case of early‐onset systemic lupus erythematosus. Due to a genetic C1qA deficiency she has been considered for the transplant list for hematopoietic stem cell transplantation. She is the only child in the family. Yet, there is no record of immunodeficiency or autoimmune disorders among parents. Since birth, the patient has shown a pattern of recurring infections, marked by prolonged and intense upper respiratory tract infections that have taken a long time to resolve, even with conventional treatments. Significantly, throughout the teething stages, she endured instances of high fever along with painful oral infections and ulcers, necessitating prolonged antibiotic treatment for their resolution. At the age of 6 months, the patient experienced a widespread and intense malar skin rash affecting the face, hands, and feet. These rashes did not respond to standard topical anti‐inflammatory treatments, such as corticosteroids and calcineurin inhibitor creams, and lasted for more than 2 months. The rashes were also sensitive to light, worsening when exposed to sunlight. Because the cutaneous findings were refractory and photosensitive, the patient was referred to a pediatric rheumatology consultant for evaluation.

**Figure 1:**
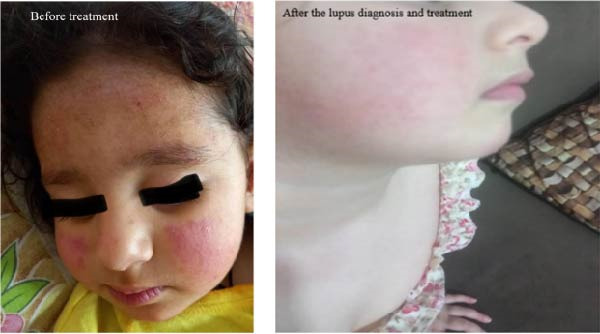
Treatment with systemic corticosteroid (prednisolone) and hydroxychloroquine resulted in significant improvement in skin lesions.

Positive laboratory tests for ANA (Table 1) and clinical history were consistent with early‐onset lupus erythematosus. Based on these findings, a diagnosis of juvenile‐onset lupus was established. Treatment with systemic corticosteroid (prednisolone) and hydroxychloroquine was promptly initiated, resulting in significant improvement in skin lesions (Figure 1). The patient also presented a history of food allergy, notably to tomato and eggs. She did not exhibit neurological symptoms or arthritis. It is also noted that she successfully received all routine childhood vaccinations by the national immunization schedule, demonstrating appropriate immune responses without exhibiting adverse reactions. Because of the history of recurrent infections in the patient’s early life, a deficiency in immune cells was suspected. Thus, immunophenotyping was performed for detecting any defects in immune cell production or maintenance in the patient. Importantly, flow cytometric analysis demonstrated normal T, B, and NK cell subsets (Table 2). Over the past year, approximately every 2 months, the patient underwent laboratory evaluations, which identified a consistent pattern of hematological and immunological irregularities. Results are indicated in Table 1. It is to be noted; however, that the results are from the time when the patient was under immunosuppressive treatment. A significant observation was the ongoing leukopenia, with WBC counts persistently below the normal range. Since lymphocyte counts were normal, the reduction in leukocytes was principally in that of neutrophils. In contrast, both the RBC and platelet counts remained stable and well within normal limits throughout the monitoring period (Table 1).

**Table 2:** Summary of flow cytometry findings of the patient’s lymphocytes in comparison to reference ranges.

CD Marker	Description	Result (relative %)	Result (absolute/μL)	Reference (aelative %)	Reference (absolute/μL)
Lymphocytes (44%)					
CD3	Pan T Cell Ag	67.51	1342.77	56–75	1400–3700
CD4	Helper T Cell (Th)	44.68	888.68	28–47	700–2200
CD8	Cytotoxic T Cell (Tc)	18.89	375.72	15–30	490–1300
CD4/CD8 Ratio	Th/Tc Ratio	2.36	—	—	—
CD19	B Cells/Most plasma cells	19.77	393.22	14–33	390–1400
CD20	B Cells/Acquired during maturation	18.74	372.73	14–33	390–1400
CD16	NK cells/Fc gamma receptor III	7.45	—	5–19	—
CD56	NK cells	6.93	—	5–19	—
CD16/56	NK cells	5.81	115.56	4–17	130–720

## 4. Results

Inflammatory markers were thoroughly evaluated, revealing a consistently elevated ESR suggesting chronic systemic inflammation. However, CRP levels were persistently low across all assessments, indicating a nonacute inflammatory state. Although serum immunoglobulin levels generally fell within normal ranges, concentrations of IgG and IgE were often near or slightly above the upper limit of the normal range. This suggested a possible trend towards hypergammaglobulinemia and potential allergic sensitization.

Liver function tests indicated variable but predominantly elevated levels of AST and ALT, with values of 41 U/L and 47 U/L, respectively (normal range: up to 30 U/L). Renal tests, which encompassed BUN, serum creatinine, and urine protein levels, consistently reflected a maintained kidney function, showing no evidence of proteinuria or compromised kidney function.

An in‐depth evaluation of autoantibody profiles indicated a distinct serological pattern. ANA and anti‐SSA tests were constantly positive, but anti‐dsDNA, anti‐CENP‐B, anti‐Jo‐1, anti‐SSB, anti‐cardiolipin IgG and IgM, anti‐beta‐2 glycoprotein I IgG and IgM, and anti‐RNP antibodies remained within normal limits (Table 1). The ANA titers were significantly elevated, more than three‐fold higher than the top limit of normal. Intriguingly, anti‐SSA antibodies were strongly positive with a titer of 89 units, exceeding the normal range of 0–5 units (Table 1) and surpassing the critical clinical threshold of 50 units, indicating significant serological activity associated with systemic lupus erythematosus. Furthermore, RF levels were substantially elevated, ranging between 55.2 and 133 IU/mL (normal: <14 IU/mL), with the most recent value of 133 IU/mL reported in Table 1, confirming marked serological activity.

**Figure 2:**
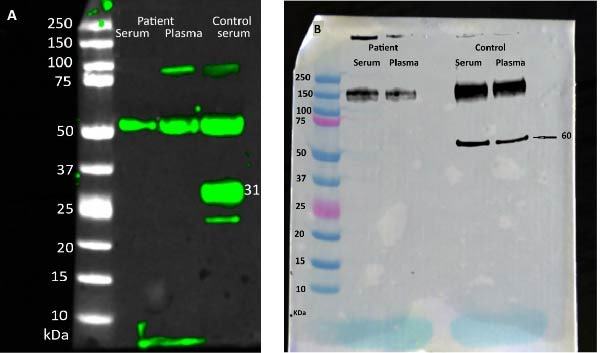
Western blot analysis of a C1qA‐deficient patient compared to a healthy control. Western blot analysis showing altered C1q protein patterns in serum and plasma from a C1qA‐deficient patient compared to normal human serum and plasma. (A) Reduced: A subunit of C1q in normal serum (31KD) were not seen in the patient, CC subunits of C1q in the serum and plasma (52KD) of patient and control. (B) NonReduced: AB subunits of C1q in normal serum and plasma (60KD) were not seen in the patient.

The complement system was also evaluated. An ELISA assay revealed slightly elevated apparent serum C1q levels. However, this does not indicate the levels of intact C1q, because by immunoblotting, fragmentation of the protein was observed (Figure 2A). The CH50 assay indicated a marked reduction in total complement activity, confirming a considerable impairment of the classical complement pathway (Table 1).

Genetic analysis was conducted to ascertain the underlying cause of the patient’s early‐onset SLE and her complement deficiency. Utilizing NGS, a homozygous pathogenic variant was identified in the C1qA gene (NM_015991.4: exon 3, c.622C >*T*, p. Gln208 ^∗^), situated on chromosome 1 (chr1:22965784 C >T). This particular nonsense mutation induces a premature stop codon at amino acid position 208, converting a glutamine codon into a stop codon. According to the guidelines provided by the American College of Medical Genetics and Genomics (ACMG), this variant has been classified as pathogenic and is linked to C1q deficiency type 1, recognized as an autosomal recessive disorder. This variant thus results in the functional impairment of the C1qA protein subunit. The detection of this homozygous nonsense mutation in the *C1QA* gene substantiates the diagnosis of hereditary C1q deficiency, elucidating the cause of the patient’s early‐onset SLE and corresponding clinical manifestation.

The abdominal and pelvic ultrasonography results were normal, showing no abnormalities. An ophthalmologic examination confirmed that the macula and optic nerve appeared healthy, with no signs of inflammation. While the neutrophil levels were decreased, flow cytometric analysis of peripheral blood lymphocytes revealed a standard distribution and proportion of T cell subsets, B cells and NK cells, indicating intact cellular immunity without any abnormalities in identified lymphocyte subsets.

### 4.1. Altered C1q Protein Pattern in Serum and Plasma

As illustrated in Figure 2A distinct difference was observed in a protein band migrating at ~30 kDa under reducing conditions. This band corresponds to the A chain of C1q, confirming its absence from the patient sample but presence in the control sample. In Figure 2B distinct difference is evident in a protein band migrating at ~60 kDa under nonreducing conditions. This band corresponds to the heterodimeric assembly of the A and B chains of C1q, indicating that the dimer remains intact in the absence of a reducing agent. The presence of this band under nonreducing conditions confirms that the interchain disulfide bonds are preserved, thereby maintaining the structural integrity of the C1q AB dimer.

## 5. Discussion

Our patient case highlights the link between complement deficiency and autoimmunity. The patient’s symptoms, recurrent infections, malar rash, oral ulcers, and leukopenia are characteristic of hereditary C1q deficiency‐associated lupus, which typically presents earlier and with more severe skin involvement than sporadic SLE [8]. The mechanism involves impaired clearance of apoptotic cells, chromatin, and immune complexes due to missing C1q, leading to persistent autoantigen exposure, breakdown of self‐tolerance and systemic autoimmunity [9].

The patient’s rapid improvement on standard lupus therapy (corticosteroids and hydroxychloroquine) confirms the inflammatory nature of the condition [10]. Genetic testing identified a homozygous nonsense mutation in C1QA, which prevents the formation of C1q AB dimers and ABC trimers. According to the ACMG criteria, the variant is classified as pathogenic and is causally associated with type 1 hereditary C1q deficiency, an autosomal recessive monogenic cause of lupus. Despite the autoimmune manifestations, cellular immunity remained intact, as demonstrated by normal T, B, and NK cell subsets, along with appropriate responses to routine vaccinations and a history of food allergies. These findings are consistent with previous reports showing that C1q deficiency primarily affects complement‐mediated immune functions without impairing lymphocyte development or cellular immune function [11]. Laboratory findings also revealed persistent decline in WBC, possibly related to lack of complement‐mediated recruitment, immune‐mediated destruction or bone marrow suppression from chronic inflammation and autoantibodies. This result is in the line of a previous study [12]. Additionally, persistently elevated ESR with normal CRP suggested a chronic low‐grade inflammatory state associated with complement deficiency. This finding is also in accordance of the Japanese report [11]. The serological profile in C1q deficiency associated lupus differs from classical SLE and is characterized by markedly elevated ANA titers, strong anti‐SSA positivity, and high RF levels, as observed in this patient. The absence of anti‐dsDNA antibodies, together with normal C3 and C4 levels but severely reduced CH50, further distinguishes monogenic lupus due to C1q deficiency from classical SLE, where anti‐dsDNA antibodies and hypocomplementemia are more typical [11]. These findings reflect the distinct immunological dysfunction associated with C1q deficiency. Elevated AST and ALT levels may indicate mild hepatic involvement related to chronic inflammation or autoimmune hepatitis in lupus, but they could also result from hepatotoxic effects of immunosuppressive therapy, including corticosteroids and hydroxychloroquine used in this case. Therefore, ongoing monitoring is required to differentiate disease‐related hepatic injury from drug toxicity [13]. Despite the profound complement defect, renal function remained normal with no evidence of lupus nephritis, suggesting that the disease is currently limited to cutaneous and hematological manifestations, consistent with early‐stage lupus in the context of C1q deficiency [14]. This is also consistent with reports that lupus associated with C1q deficiency may present as predominantly a mucocutaneous, hematological disorder initially, before renal manifestations of lupus arise [15]. However, the need for long‐term follow‐up is important, as renal involvement may develop with time.

## 6. Conclusion

This case highlights the critical role of C1q in maintaining immune homeostasis and preventing lupus in the pediatric population. It underscores a key clinical observation: in children presenting with early‐onset, severe, cutaneous, and treatment‐resistant lupus, genetic testing for complement deficiencies should be considered. Our multidisciplinary approach, including clinical evaluation, serologic testing, genetic mutation analysis, and protein analysis (Western blot), provided a comprehensive understanding of the patient’s disease pathogenesis. Despite ongoing disease activity and the presence of a genetic complement deficiency, hematopoietic stem cell transplantation remains a potentially curative option. This reinforces the importance of accurate and timely molecular diagnosis in cases of monogenic lupus.

## Author Contributions

Andisheh Mosaffa Jahromi, Hadi Mottaghipisheh, and Fatemeh Mirzaei wrote the manuscript. Seppo Meri and Kurosh Kalantar interpreted the data. Andisheh Mosaffa Jahromi, Hadi Mottaghipisheh, and Kurosh Kalantar analyzed peripheral immune phenotyping. SM analyzed the interpretation of complement protein levels. Hadi Mottaghipisheh, Kurosh Kalantar, Fatemeh Mirzaei, and Seppo Meri conceived and coordinated the study, interpreted the data, and co‐wrote the manuscript. All authors discussed the results and commented on the manuscript.

## Funding

This research was funded by the Shiraz University of Medical Sciences (Grant 33359) and was awarded to Kurosh Kalantar.

## Ethics Statement

This study was approved by the Ethics Committee of Shiraz University of Medical Science (Number 33359)

## Consent

Written informed consent for publication of the child’s clinical details and any accompanying images was obtained from the patient’s parents, who are the legal guardians.

## Conflicts of Interest

The authors declare no conflicts of interest.

## Data Availability

The clinical data used to support the findings of this study are included within the article.
